# The Additional Diagnostic Value of the Three-dimensional Volume Rendering Imaging in Routine Radiology Practice

**DOI:** 10.7759/cureus.5579

**Published:** 2019-09-05

**Authors:** Alper H Duran, Munevver N Duran, Irfan Masood, Lynsey M Maciolek, Huda Hussain

**Affiliations:** 1 Radiology, Mount Sinai West, New York, USA; 2 Radiology, Rice University, Houston, USA; 3 Radiology, University of Texas Medical Branch, Galveston, USA

**Keywords:** high resolution computed tomography (hrct), maximum intensity projections, magnetic resonance angiography, mip, vascular anatomy, volume rendering, surgical planning, ct angiography (cta), three-dimensional, 3dvr

## Abstract

Three-dimensional volume rendering (3DVR) is useful in a wide variety of medical-imaging applications. The increasingly advanced capabilities of CT and MRI to acquire volumetric data sets with isotropic voxels have resulted in the increased use of the 3DVR techniques for clinical applications. The two most commonly used techniques are the maximum intensity projection (MIP) and, more recently, 3DVR. Several kinds of medical imaging data could be reconstructed for 3D display, including CT, MRI, and ultrasonography (US). In particular, the 3D CT imaging has been developed, improved, and widely used of late. Understanding the mechanisms of 3DVR is essential for the accurate evaluation of the resulting images. Although further research is required to detect the efficiency of 3DVR in radiological applications, with wider availability and improved diagnostic performance, 3DVR is likely to enjoy widespread acceptance in the radiology practice going forward.

## Introduction and background

As medical-imaging devices such as CT and MRI scanners continue to develop, we can now observe the human body in increasing detail. The human body can now be observed in 3D space by visualizing 3D medical images, and this technique now plays an important role in clinical applications [1, 2]. The three-dimensional volume rendering (3DVR) imaging is now a major area of clinical and radiological practice. It is proving to be far more than just a solution in diagnostic dilemmas. One of the great advantages of 3DVR is that it can provide all the necessary information in a single radiologic study in cases that previously required two or more studies [3]. 3DVR also allows the radiologist and clinician to address specific questions concerning patient care by enabling them to interactively explore the different aspects of the data imaging set. In contrast to the growing problem of information overload presented by the large acquisition rates of modern scanners, 3DVR has the potential to simplify the standard radiologic study. All 3D-rendering techniques reveal a 3D volume of data in one or more two-dimensional (2D) planes, conveying the spatial relationships inherent in data with the use of visual depth cues [4, 5]. 3DVR is an alternative that has inherent advantages over the more commonly used projectional techniques. An additional advantage of the VR technique, one it shares with surface displays, is that it allows for perspective rendering [2]. Over the past decade, computer systems have become both economical and more powerful. Thus, the 3DVR modality is projected to become economically feasible in the near future [3-5]. In this review, we present a brief history of the 3DVR technique and emphasize the potential diagnostic contributions of the the 3DVR imaging modality.

## Review

The first implementation of the VR technique grew out of research done at the Mayo Clinic, Minnesota, US, in the 1970s. Advances in the image processing hardware and the integration of new data manipulation techniques led to parallel developments in VR techniques at the University of North Carolina [2]. In the 1980s, the major advancement in the field was the refinement of CT technology to the point where radiologists could obtain sub-second scans with 0.3 mm resolution and the maturation of MRI [4, 6]. After the 1990s, radiologists predicted that one of the major achievements of that decade would be the widespread adoption of the computed image-processing technology by the medical community. The 3D imaging reformats conventional imaging data into a series of images that closely resemble the original studied structure. 3DVR is becoming a valuable tool for both the diagnostic and therapeutic display of digital information [5, 6]. Recently, many of our clinicians in radiation therapy and oncology, orthopedics, and surgery have adapted aspects of this technology for their own specific applications. It is imperative that radiologists become actively involved in this newer imaging technology or, alternatively, run the risk of being left behind by clinicians [6, 7].

The three-dimensional volume rendering (3DVR) technique and maximum intensity projection technique (MIP)

The 3D-imaging techniques like CT and MRI are entirely dependent on computer systems. This can be linked directly to the evolution of computer hardware [3, 7]. The 3DVR technique takes the entire volume of data, shows the contributions of each voxel along a line from the viewer's eye through the data set, and displays the resulting composite for each pixel of the display [4, 8]. The VR technique has been implemented both in specialized hardware and software for more readily available systems. The software applications potentially benefit from being loosely tied to the computer platform. A new generation of parallel processing computers is now commercially available, which portends even more advantages for VR in hardware. Thus, 3D images can be rendered in real-time at rates exceeding 5-10 frames/sec. The incorporation of information from the entire volume can lead to greater fidelity of data; however, more powerful computers are required to perform VR modality at a reasonable speed [4, 8, 9]. We think that the broader availability of computer systems will allow for the widespread use of 3DVR in routine radiology practice in the near future.

The maximum intensity projection (MIP) technique is a 3DVR method that evaluates each voxel along a line from the viewer's eye through the volume of data and selects the maximum voxel value, which is then used as the displayed value. The clinical utility of MIP images has been assessed extensively, and the MIP technique has proved to be particularly useful in its original application in creating angiography images from CT and MR series [2, 4, 10]. Schriener et al. showed that different versions of the MIP algorithm could produce very different images. Thus, MIP and VR should be thought of as families of related image-processing techniques rather than as independent entities [11]. The MIP technique has several related artifacts; MIP images are typically not displayed with surface shading or other depth cues, which can make an assessment of 3D relationships difficult. At the same time, volume averaging coupled with the MIP algorithm commonly leads to MIP artifacts. For instance, a normal small vessel may have "string of beads" appearance because it is only partially represented by voxels along its length [4, 11].

Three-dimensional volume rendering (3DVR) imaging applications in radiology practice

There are many applications for the 3DVR imaging modality in radiology practice. Perhaps, the most important long-term application is the routine reading for radiologic data. 3DVR is likely to be the one development that would affect the radiologists most profoundly in their daily practice. Indeed, the surgeons will someday have their own interactive volume displays, which suggests how the role of radiology will rest more firmly on shared resources and expanded communication. Thus, the role of 3DVR in radiology continues to grow rapidly [4, 6, 8-11].

**Figure 1:**
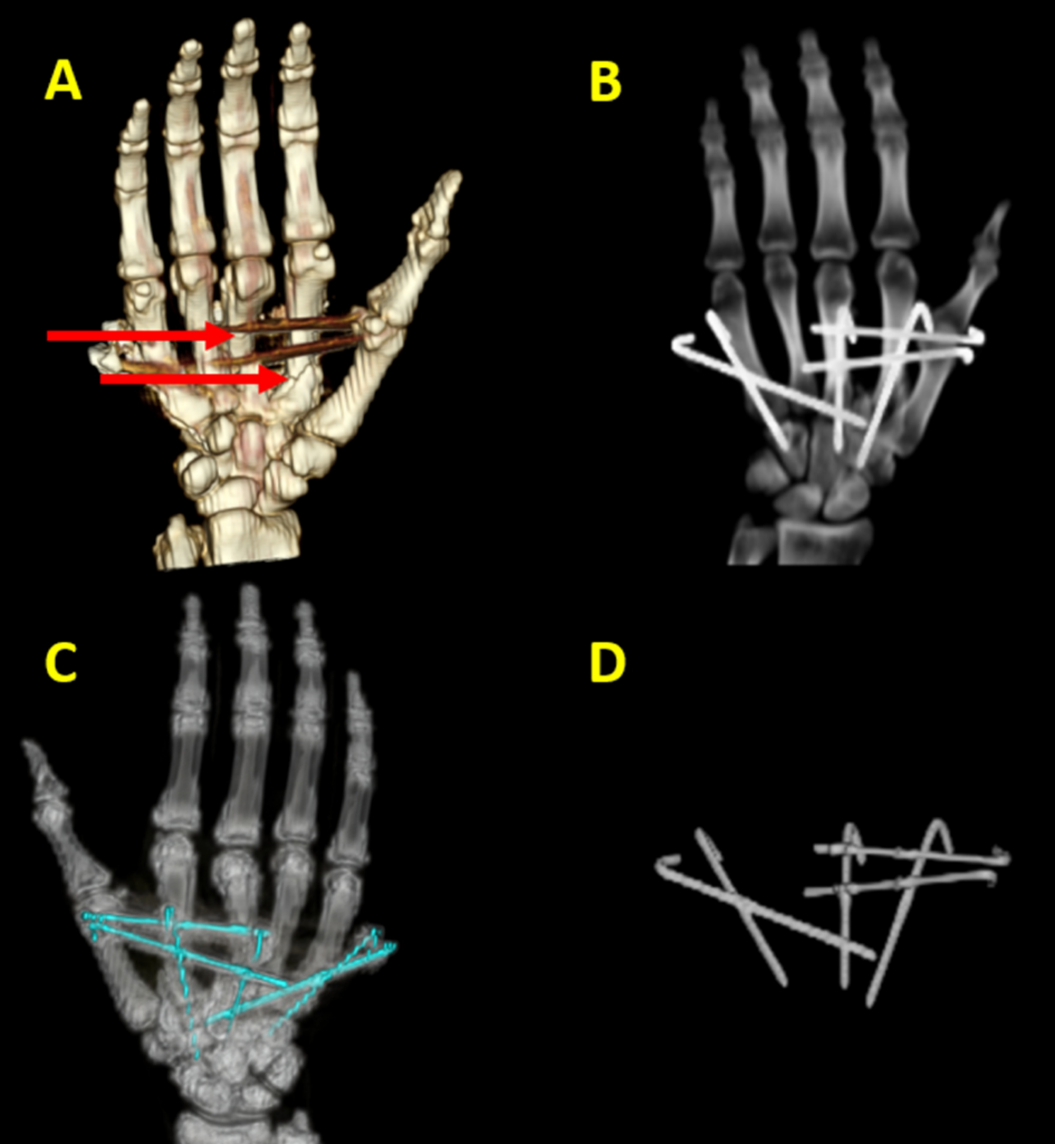
Metacarpal fracture hardware positioning in three-dimensional volume rendering (3DVR). (A) 3DVR images demonstrate multiple fixation pins and their orientation in a patient with several metacarpal fractures (red arrows). (B) The software allows for windowing that provides better visualization of the hardware in the same patient. (C) The software also allows for windowing that provides better visualization of bone structure and malunion of the fractured bone fragments. (D) Elimination of the bones and soft tissues provides an evaluation of hardware positioning and hardware migration.

One of the 3DVR-imaging applications is musculoskeletal imaging. Since the inception of 3DVR in CT protocols, applications in skeletal anatomy have provided an opportunity for demonstrating the efficacy of this new technique [4, 6]. Naraghi and Whitere have studied commonly available 3D MRI techniques and discussed the literature to-date regarding the utility of such techniques in the assessment of the internal derangement of joints. They stress that the technical advances, including higher-field-strength MRI systems, high-performance gradients, high-resolution multichannel coils, and pulse sequences with shorter acquisition times, have made 3D MRI with reasonable acquisition times feasible [12]. Additionally, acetabular fractures, pelvic ring fractures, ankle trauma, tibia plateau fractures, shoulder anomalies, soft-tissue masses, congenital anomalies, tumors, and musculoskeletal inflammatory disease can be easily evaluated via 3DVR images [10, 13]. The overview function of the 3D imaging becomes more critical as the size of the area of interest and the complexity of the trauma increases. The pelvis, for instance, is far too large to be adequately represented in any planar section. In a severely traumatized patient, 2D images detail the multiple abnormalities, but the 3D imaging integrates the many individual findings into a whole, more focused assimilation than the sum of its parts [4, 6]. 3D bone modeling has added another dimension to the surgical planning and localization of foreign bodies. Angulation or malunion of fractured bones, in addition to hardware positioning or migration, can be better evaluated by 3DVR images (Figure 1).

**Figure 2:**
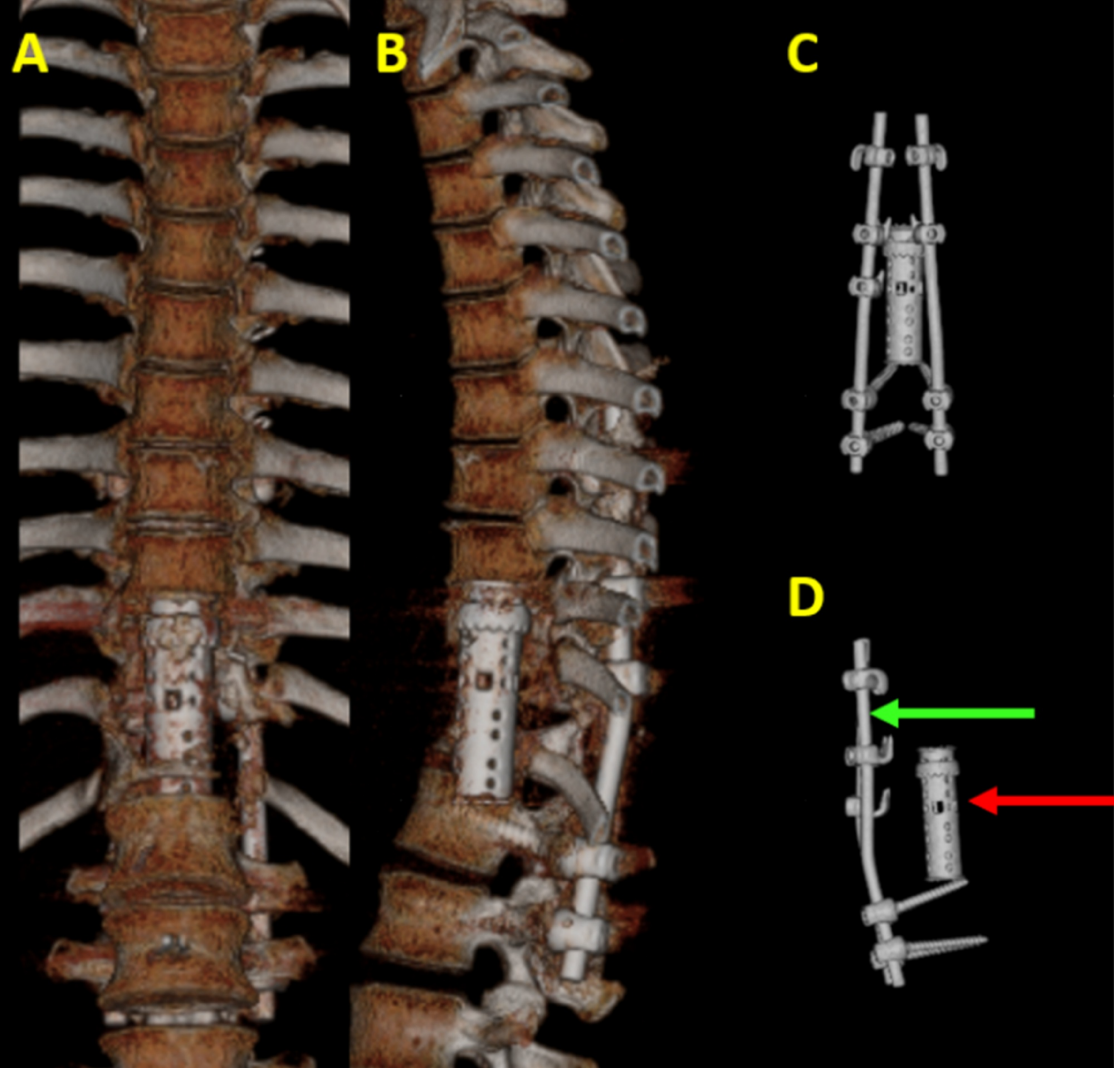
Spinal fixation hardware and disc spacer in three-dimensional volume rendering (3DVR). 3DVR images demonstrate spinal fixation hardware and a disc spacer on (A) anterior-posterior and (B) lateral views. The elimination of the surrounding soft tissue and bones allows for the better visualization of the spinal fixation hardware (green arrow) and disc spacer (red arrow) in order to evaluate for possible hardware complications on (C) anterior-posterior and (D) lateral views.

The elimination of the surrounding soft-tissue structures helps the evaluation of the hardware and its possible complications (Figure 2).

In addition to greatly improving the definition of the pathologic condition, 3DVR bone modeling may be used to perform surgical simulation procedures [13, 14].

3DVR is not confined to being used for surgical preoperative planning and intraoperatively. It can be used in postoperative patients as well. Ciodaro et al. found that the use of 3DVR based on high-resolution computed tomography (HRCT) temporal bone in postoperative patients with cochlear implants was much more accurate in detecting a representation of the topographic complex of the cochlea than HRCT itself. VR based on HRCT temporal bone enables the creation of a dataset of images that envisages the structure of the human ear in 3D. They noted that 3DVR led to better detection in images when compared with the traditional CT imaging [15].

**Figure 3:**
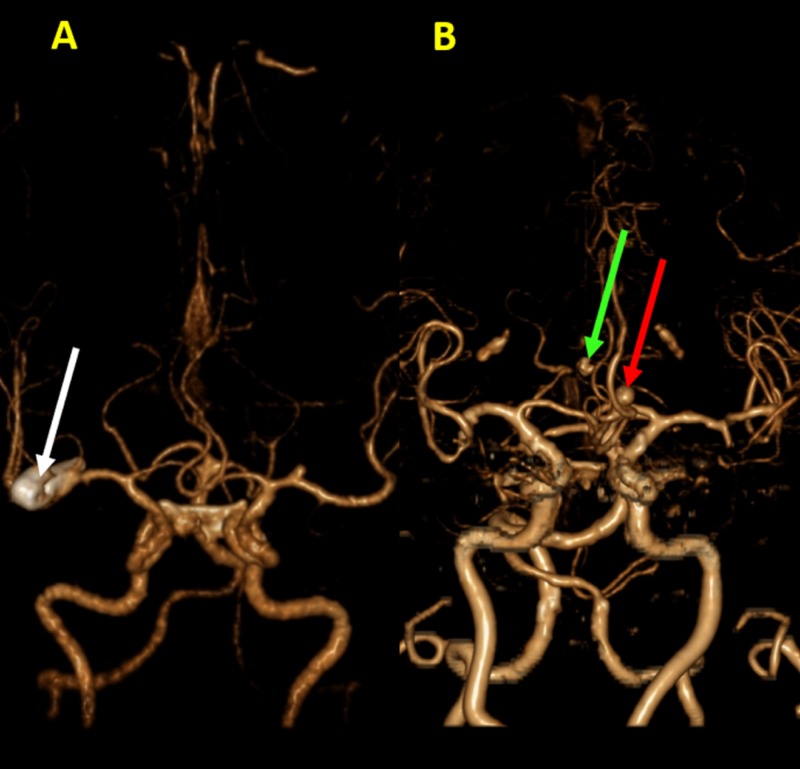
Intracranial aneurysms in 3hree-dimensional volume rendering (3DVR). 3DVR images demonstrate multiple intracranial aneurysms in two different patients. (A) There is presence of a large mixed saccular-fusiform aneurysm at the M2 segment of the right middle cerebral artery (white arrow). (B) In a separate patient, there are two subcentimeter saccular aneurysms seen at the anterior communicating artery (green arrow) and proximal left anterior cerebral artery (red arrow).

Image registration has been used to produce multimodal cross-sectional images for neurosurgical diagnosis and planning. The process of image integration was recently advanced by the construction of multimodal 3D computer models of the brain [4, 6]. 3DVR views of the brain surface demonstrate specific convolutions such as pre-central, post-central, left inferior frontal, and other gyri. 3DVR images are used to understand the spatial relationship between these gyri and brain lesions, which are visualized as distortions of the surface. 3DVR images can also reveal important preoperative information on intracranial aneurysms to the surgeons or interventional radiologists that cannot be obtained in any other way (Figure 3) [4, 16].

Craniofacial deformities are rare but complex birth defects. The diagnosis of such anomalies traditionally has been made based on the physical examination augmented by skull-imaging studies. The availability of CT images, augmented first by means of multiplanar reformatting and, later, 3D simulation, profoundly expanded imaging options for the diagnosis and therapeutic planning related to craniofacial deformities. In general, the more complex and unusual a given case, the greater the potential contribution of 3D images. The 3DVR techniques are increasingly used for assessment of the skull before craniofacial surgery [4, 6, 17, 18].

CT colonography (CTC) with 3DVR is an established test for asymptomatic individuals for early detection of colorectal cancer. Both the American College of Radiology and the American Cancer Society have approved CTC for colorectal cancer screening in patients older than 50 years, those with a positive fecal occult blood test, and individuals at moderate risk with a personal history of adenoma or colorectal cancer or with family history of adenoma or cancer in any first-degree relative [19, 20]. CTC with 3DVR enables the acquisition of multiple thin sections, superior multiplanar reconstructions, and a faster imaging time for the detection of colorectal cancer and polyps [21].

Radiation oncologists must integrate the relevant data from a daunting array of diagnostic examinations. The main aim is the complete spatial definition of the tumor and its relationship to juxtaposed normal anatomic structures. The need to superimpose and simulate radiation therapy carries the demands made on the 3DVR imaging one step further than those of diagnostic work alone. The approximated tumor volume can also be drawn on the image to begin modifying the proposed field and to define surrounding tissues at risk by 3DVR images [4, 22]. There are many different areas in oncology that can benefit from 3DVR imaging. CT with the 3DVR technique can be used to detect tumor resectability and preoperative planning for tumor resection in patients with a variety of neoplasms, such as primary and/or metastatic hepatic malignancies, renal cell carcinoma, or gastric cancer [3, 4, 6]. Recent advances in liver surgery have made the role of 3DVR modality critical to the surgeon in liver transplantation [23, 24]. In addition to 3DVR, cinematic rendering is another recently discovered technique that can aid in understanding the magnitude of disease and the planning needed for specific intervention. Cinematic rendering enables the development of images with soft-tissue details and realistic shadowing effects to represent the depth and visualize the relative positions of objects, which can be very advantageous in diagnostics and planning [25]. For instance, Rowe et al. published the first case of using 3D cinematic rendering for clear cell renal cell carcinoma, which facilitated better surgical planning. They hypothesize that a possible advantage of cinematic rendering over volumetric rendering might be the improved soft-tissue detail that can show visual textural features, which would aid in characterizing renal masses; however, further research is needed in this regard [26].

In clinical applications, it is extremely important to grasp the vessel structures correctly when planning surgical procedures or understanding a disease. This is because a completely different surgical plan may be chosen based on a slight difference in the vessel structures. Prior to laparoscopic surgery, a surgeon takes 3D CT images for laparoscopic surgical planning and observes the branching patterns of the abdominal blood vessels by performing interactive VR of 3D CT images, which show different branching patterns among patients [7, 10]. Today, CT angiography with 3DVR plays a crucial role in increasing diagnostic efficacy for cardiovascular studies. Spiral CT, in combination with 3DVR, reveals information at a lower cost than conventional angiography [1, 27, 28]. Pang et al. used motion-corrected sensitivity encoding with 3D projection reconstruction, which accelerated whole-heart coronary MR angiography procedures for obtaining good image quality [29]. The role of cardiovascular MR with 3D reconstruction is particularly important in the evaluation of recognized and unrecognized cardiovascular diseases. It also provides a noninvasive means to obtain many layers of information at a tissue level, including fibrosis, edema, and congenital cardiovascular anomalies [27-30].

**Figure 4:**
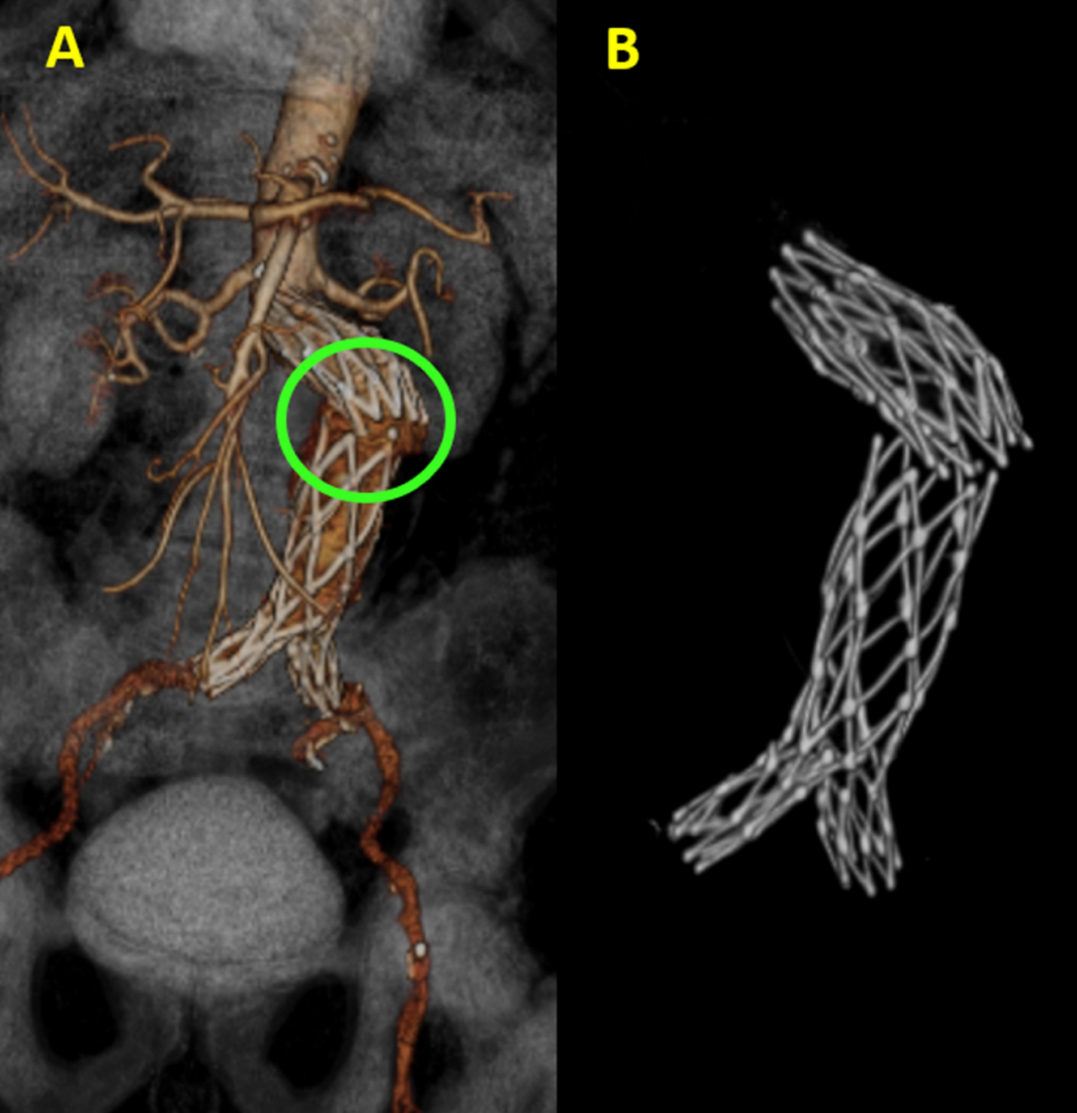
Aortic stent fracture in three-dimensional volume rendering (3DVR). (A) 3DVR images demonstrate complete transverse aortic stent fracture (green circle) with angulation and slight lateral stent displacement. (B) The elimination of the surrounding soft tissue and vessels allows for a better visualization of the hardware.

Endovascular repair of abdominal aortic aneurysms has been followed in clinical practice for more than a decade. It has been confirmed to be an effective alternative to conventional open surgery, especially in patients with co-morbid medical conditions. Helical CT angiography is the preferred imaging method in the follow-up of endovascular repair. The recent introduction of multislice CT scanners has augmented its diagnostic role in this area. The diagnostic value of multislice CT has been complemented by a series of 3D post-processing VR images, which assist the vascular surgeons in accurately assessing the effect of endovascular repair by providing additional information, when compared to conventional 2D axial images, for the evaluation of stent-grafts and their complications (Figure 4).

These reconstructions include multiplanar reformation, curved multiplanar reconstruction, shaded surface display, MIP, VR, and virtual endoscopy [31, 32].

**Figure 5:**
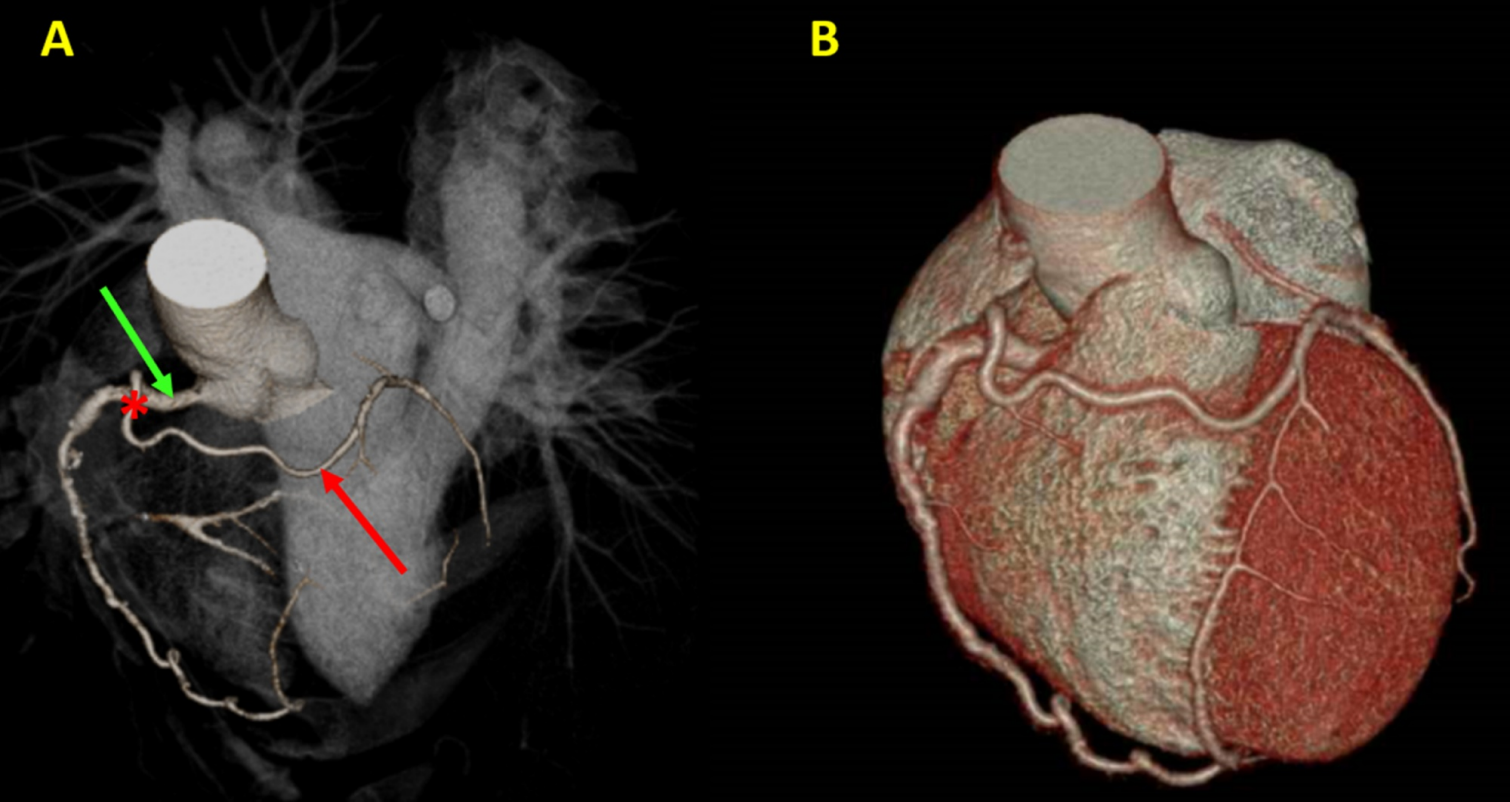
Coronary arteries in three-dimensional volume rendering (3DVR). (A) 3DVR images demonstrate the course of the left main coronary artery (red arrow) originating (red asterisk) from the right coronary artery (green arrow). (B) It is also important to correlate this coronary artery anatomy with the surrounding soft tissue to assess if there is any myocardial bridging.

CT angiography with 3DVR has the advantage of being a noninvasive examination that can reveal all the information required for many applications. The accuracy of 3DVR images in a variety of applications, such as thoracic and abdominal aneurysm, intracranial aneurysm, and coronary arterial variations (Figure 5), equals that of conventional techniques.

Multidetector CT (MDCT) with 3D reconstructions, MRI, and digital subtraction angiographies (DSA) can diagnose intracranial aneurysms. The combination of MDCT angiography with 3DVR and DSA enables the definitive diagnosis of intracranial aneurysms and associated abnormalities such as interrupted aortic arch [6, 31-33].

There are some limitations to the 3D-imaging reconstructions. VR always accurately depicts 3D relationships, while MIP may not do so, especially on arterial phase-dominant images that show both arterial and venous structures. VR not only displays the vascular anatomy but also defines the soft tissue, muscle, and bone [34, 35]. Thus, VR enables a color display, which often improves the visualization of complex anatomy and 3D relationships.

Calcifications in the vessel walls are usually more of a problem with MIP than VR. With MIP, the luminal narrowing may be overestimated, whereas, with VR, the vessel lumen and wall calcifications can usually be individually defined [4, 36]. MIP may allow the visualization of the smaller branch vessels with less work than is required for VR. The capability of showing only the brightest pixels may help to define smaller branch vessels in the liver, kidneys, and lungs [37]. To summarize, VR and MIP images, when evaluated together, enable a comprehensive understanding of the full extent of a pathologic process. VR also provides additional information beyond the vascular map [4, 6, 36].

## Conclusions

In conclusion, 3DVR imaging is a flexible technique with a high screen resolution. It can help the radiologists more effectively interpret the large volumes of data generated by modern scanners. To obtain accurate results and reliable diagnoses, the 3DVR technique should be added into routine radiology practice. We recommend the addition of the 3DVR imaging to provide better image quality in routine radiology practice.
